# Extended Exposure to Alzheimer's Risk Factors Increases Alzheimer's Diagnosis Risk Amplified in APOε4 Carriers: Implication for Delayed Onset of Alzheimer's Risk Factors

**DOI:** 10.1002/alz.091806

**Published:** 2025-01-09

**Authors:** Simona Merlini, Francesca Vitali, Roberta Diaz Brinton

**Affiliations:** ^1^ University of Arizona, Tucson, AZ USA

## Abstract

**Background:**

Obesity, diabetes, hypertension, hyperlipidemia, and depression are relevant Alzheimer’s Disease (AD) modifiable risk factors (RFs). However, little is known about how the duration of these conditions, influenced by unmodifiable AD risk factors, such as chromosomal sex and APOE genotype, affects the associated risk. Modeling interactions between patient’s temporal and clinical patterns, integrated with the probability of developing AD remains challenging, particularly when considering the multifactorial progression of AD. To address these challenges, we conducted a retrospective analysis using longitudinal data from the UK Biobank.

**Methods:**

Inclusion criteria were age older than 55 years, no prior history of neurodegenerative disease, neurosurgery, or cancer, and enrollment with at least 3 years of follow‐up. Propensity score matching was performed based on age at recruitment, educational level, center, and Charlson comorbidity index. Extended Cox proportional hazard models (CPHMs) for time‐to‐event prediction were used to evaluate the impact of the age‐specific effect of multiple AD‐RFs, in combination with sex and APOEε4 carrier status. Age stratification was evaluated when a violation of the hazard ratio (HR) proportionality assumption occurred during aging.

**Result:**

Preliminary findings revealed RF‐specific differences in AD on‐set based on the age of RF diagnosis. After age stratification, the risk of developing AD was significantly greater than the APOEε4 effect if hypertension and diabetes were diagnosed before 62yo, while if diagnosed after 72yo, APOEε4 was the major contributor to increased AD risk. Obesity had a 54% increased risk of developing AD if diagnosed before 62yo and was associated with nearly 3 times increased risk if diagnosed between 62‐72yo. Lastly, hyperlipidemia and depression were associated with an age‐strata‐independent increased AD risk of 33% and 69%, respectively.

**Conclusions:**

This study identified critical tipping points indicating a decline in the hazard ratio of modifiable RFs with aging and a stronger association of APOEε4 with late‐onset AD. Age stratification within CPHMs provided valuable insights into age‐specific hazard ratios and identified age‐dependent RFs. Furthermore, these results highlight interactions between age, sex, and APOE genotype which could inform a precision medicine approach for AD prevention.

